# Delayed Interval Delivery in Preterm Premature Rupture of Membranes in Dichorionic Triamniotic Triplets: Ethical Considerations for Maternal Health Case Report

**DOI:** 10.1155/2022/4766523

**Published:** 2022-07-21

**Authors:** Ali Hosiani, James Brown, Indika T. Alahakoon

**Affiliations:** ^1^Department of Obstetrics & Gynaecology, Blacktown Hospital, Blacktown, New South Wales, Australia; ^2^Department of Maternal Foetal Medicine, Westmead Hospital, Westmead, New South Wales, Australia

## Abstract

**Background:**

Although there are numerous studies on delayed interval delivery in twins, this study is one of few reporting on preterm premature rupture of membranes in triplets and even fewer on dichorionic triamniotic triplet twins. The case presented here highlights the important interplay between informed consent and patient autonomy. *Case Presentation*. A 37-year-old woman gravida 1, para 0 with a dichorionic triamniotic triplet pregnancy experienced preterm premature rupture of membranes of the singleton triplet at 15 weeks and six days of gestation. Delayed interval delivery was offered to the parents, who chose to continue the pregnancy while acknowledging the risks for maternal and foetal health. The patient was treated with prophylactic intravenous antibiotics and discharged on oral antibiotics after an eight-day admission. Two days after being discharged, she was readmitted with clinical signs of chorioamnionitis. Within six hours, the preterm premature rupture of membranes singleton was delivered. Three days later, she again presented to the hospital with preterm premature rupture of membranes of one of the dichorionic twins. After discussion with the maternal foetal medicine team, the parents chose to terminate the pregnancy. Delayed interval delivery was not successful in this patient, and it is unclear at which gestational age it is too early to offer expectant management.

**Conclusions:**

The case affirmed the very poor foetal survival rate when the first delivery occurs at under 20 weeks' gestation. A standardised management of delayed interval delivery should be established to assist with consistent parental counselling.

## 1. Introduction

Multiple pregnancies carry greater risks to maternal and foetal health than singleton pregnancies [1]. These risks include miscarriage, preterm premature rupture of membranes (PPROM), and preterm birth. PPROM complicates 3% of all pregnancies and is associated with up to 30% of preterm births [2]. Up to 36% of twin and 28% of triplet pregnancies are affected by PPROM before 28 weeks of gestation [3, 4]. The incidence of PPROM has increased in the past decade, primarily due to the increased use of assisted reproductive technologies [5].

## 2. Case Presentation

A 37-year-old woman (gravida 1, para 0) became pregnant with dichorionic triamniotic (DCTA) triplets following the transfer of two fresh embryos. She underwent in vitro fertilisation (IVF) due to a history of grade 4 endometriosis. The patient's treatment for endometriosis had included multiple laparoscopic surgeries: a left salpingectomy, a small bowel resection, and a stent insertion for a left ureteric stricture. An early dating scan at eight weeks and two days of gestation confirmed that foetus A was a singleton while foetuses B and C were diamniotic twins. The foetal biometry of triplet B was smaller by four days compared to the other two foetuses. Her antenatal serology was unremarkable except for subclinical hypothyroidism and vitamin D deficiency. She was managed in a tertiary maternal foetal medicine unit from 12 weeks' gestation.

The patient was counselled about the foetal and maternal risks of a triplet pregnancy and was given the option of selective foetal reduction. An early structural scan at 12 weeks' gestation was unremarkable. After the scan, she continued to take early pregnancy progesterone pessaries (200 mg nocte) and aspirin (150 mg daily), in addition to pregnancy multivitamins.

At 15 weeks and 6 days of gestation, the patient presented to hospital with spontaneous PPROM of the singleton pregnancy. She was admitted and commenced on intravenous ampicillin and gentamicin. The couple were extensively counselled about the poor prognosis of her pregnancy with PPROM at such early gestation and the possible poor maternal sequala if she developed chorioamnionitis. Given her history of infertility, they decided to continue with conservative management. After an eight-day admission, the patient was sent home on oral phenoxymethylpenicillin. The patient's progress was monitored by measuring her inflammatory markers weekly (as an outpatient) and her temperature daily, and the foetal heart rate was monitored regularly.

Two days after discharge, at 17 weeks and 3 days of gestation, she represented to the emergency department with increased vaginal spotting and discharge. She was tachycardic, and her inflammatory markers were increased. She was diagnosed with chorioamnionitis. After multiple vasovagal episodes, a speculum revealed an open cervix with a foot in the vagina. The foetus was delivered manually, with the placenta left in situ, and the umbilical cord was clamped and left in the vaginal vault.

Ultrasound assessment at this stage showed a viable monochorionic diamniotic (MCDA) twin pregnancy with foetus B estimated to weigh in the 7th centile and foetus C on the 16th centile. Although there was some disparity in growth, no evidence of twin-to-twin transfusion syndrome was observed. The patient was again managed as an outpatient and monitored for signs of chorioamnionitis. Rotating antibiotics were commenced, alternating between erythromycin 500 mg BD and clindamycin 300 mg BD to reduce the risk of antimicrobial resistance.

At 18 weeks and three days of gestation, the patient represented with PPROM of one MCDA twin. Despite initial intravenous antibiotic therapy, the patient had ongoing signs of sepsis: febrile, hypotensive, and tachycardic. The parents were counselled about the worsening maternal condition secondary to chorioamnionitis. This was the fourth and final counselling session for the pregnancy, and they chose to proceed with induction of labour at 18 weeks and three days of gestation. The partner was always supportive of the patient's decisions. The delivery was uneventful.

Histopathology of the two separate placental discs revealed necrotising chorioamnionitis, a sign of maternal inflammatory response. No foetal response was observed in all three umbilical cords.

The patient was followed up in the preconception clinic four months after the delivery of her remaining twins. She was keen to try another cycle of IVF using her stored frozen embryos. It was recommended that she only have one embryo transferred in future, to avoid the complications she experienced in her previous pregnancy. The patient was advised that the outcome of her previous pregnancy was likely due to the multiple embryo transfer and resulting triplet pregnancy.

## 3. Discussion

This study illustrates a rare case of failed DID in a DCTA pregnancy affected by PPROM in the second trimester. The patient's willingness to persevere despite potential severe maternal morbidity reflects a highly valued pregnancy after a long-standing history of complicated primary infertility. The events raise important questions about the ethics of avoidable maternal sequelae in pregnancies unlikely to result in a good long-term outcome.

### 3.1. Maternal Morbidity and Delayed Interval Delivery

Maternal morbidity is common with DID. Up to half of the documented patients suffer intrauterine infections leading to fulminant maternal sepsis [5, 6].

A systematic review conducted by Cheung et al. included three cases of DCTA triplets [1]. The study concluded that DID is associated with improved perinatal outcomes for those second twins/triplet pregnancies when the first twin is delivered between 20 and 29 weeks [1]. A prospective cohort study reported on one DCTA triplet pregnancy, which was delayed by only five days. However, the mean delay in all the triplet pregnancies was 18 days in comparison to 19 days in twins [3]. There were 12 triplet pregnancies in the cohort study, among which seven triplets were born before 25 weeks. None of the initial foetuses survived, and only two remaining triplets from the same pregnancy survived with a DID of 118 days [1]. However, these patients had received tocolysis as part of their DID protocol.

Our patient was 15 weeks and six days of gestation when she experienced PPROM of the singleton triplet. This rupture occurred in the previable period. The literature indicates that for DID to be considered, the following conditions should not be present: nonreassuring foetal status, congenital abnormalities, rupture of membranes of the remaining foetus, chorioamnionitis, and/or severe haemorrhage, which is a sign of placental abruption [3]. This case did not have any of these conditions initially and was, therefore, deemed appropriate for DID.

### 3.2. Patient Factors and Decision-Making in Delayed Interval Delivery

Despite the patient being at previable gestation and the literature reporting survival of the remaining foetuses to be as low as 29% [1], other factors were considered in the decision-making process. The patient was a primiparous woman with an IVF pregnancy and a significant history of endometriosis requiring multiple laparoscopic procedures to correct her pathology. These factors undoubtedly influenced the parents' decision to continue the pregnancy despite extensive counselling on the maternal risks. There are documented cases of similar situations where one or more of the babies has reached viability.

Wooldridge et al. reported on a trichorionic triamniotic pregnancy of a 27-year-old woman who experienced a miscarriage of one triplet at home. The patient was given a rescue cerclage and treated with antibiotics and daily progesterone suppositories until delivery. She delivered two babies at 31 weeks' gestation. The babies were in good condition but required ventilatory support for 72 hours. There were no specific maternal complications [7].

Ghorbani and Moghadam reported on a PPROM and delivery of a foetus in a triplet pregnancy despite a cerclage being performed early in the pregnancy. The placenta did not expel spontaneously and was left in situ. The patient was discharged following administration of prophylactic antibiotics and tocolysis. She subsequently gave birth via caesarean section at 33 weeks and four days of gestation to two babies with good agars but who required admission to the neonatal intensive care unit [8].

Therefore, it appears the choice to offer conservative management throughout the pregnancy was appropriately informed by the published literature. The woman and her partner were counselled at many stages of the pregnancy and given the opportunity to think of their options and ask questions. The partner was always very supportive of the patient's decisions and actively involved in the discussions.

### 3.3. Antibiotic Choice in Delayed Interval Delivery

After the delivery of the singleton triplet, the patient was treated with the same antibiotics mentioned above. There is no published recommended routine antibiotic regimen, duration, or route, but most clinicians have opted for administration of prophylactic therapy [5, 6, 9, 10]. Most of the antibiotics of choice were the same as those typically used for PPROM, as it is believed that the cause and route of infections are essentially the same [5].

When the patient was stepped down to rotating erythromycin and clindamycin, this was an improvised approach followed in the absence of standard protocols for antimicrobials in DID. Our centre used rotating antibiotics as a standard treatment for PPROM, which was done to prevent antibiotic resistance [11].

### 3.4. Delayed Interval Delivery and Cervical Cerclage

A cervical cerclage was not performed in this case as an ultrasound showed the cervix to be long and closed. The idea behind cerclage in DID is that it can only stabilise the cervix but minimise exposure of the foetal membranes to vaginal bacteria [12]. Some authors have opted for routine cervical cerclage [5], while others have not [5, 12–14]. The idea of cerclage is controversial without cervical incompetence as some suggest that the actual procedure puts the patient at risk of further infection and can contribute to the rupture of membranes of the remaining foetus [15]. As there is no consensus or adequate evidence on the application of cervical cerclage, it is difficult to determine if having performed it routinely in the patient, who did not show evidence of cervical incompetence and was on progesterone, would have resulted in an alternative outcome. However, it was an appropriate decision to not perform cervical cerclage when the patient presented with PPROM of one MCDA twin only seven days after delivering the first triplet.

A study on obstetric DID with and without cerclage in twin and triplet pregnancies found that cerclage may provide a longer interval. However, there is no consensus on the best treatment protocol, and each case should be evaluated individually [16].

## 4. Conclusion

Although there are numerous studies on PPROM in twins, this study is one of few reporting on PPROM in triplets and even fewer on DCTA twins. None of the triplets survived, and the delay was short-lived, lasting seven days, limited by the reoccurrence of PPROM in the remaining triplet. The case affirmed the very poor foetal survival rate (29%) when the first delivery occurs at under 20 weeks' gestation.

The case presented here highlights the important interplay between informed consent and patient autonomy. Throughout the pregnancy, the patient was included in therapeutic discussions and, ultimately, chose the course of action that she thought was best for her and her pregnancy.

## Abbreviations

PPROM: Preterm premature rupture of membranes
DID: Delayed interval delivery
DCTA triplets: Dichorionic triamniotic
MCDA: Monochorionic diamniotic
IVF: In vitro fertilisation.
